# Meiotic gatekeeper STRA8 regulates cell cycle by interacting with SETD8 during spermatogenesis

**DOI:** 10.1111/jcmm.15080

**Published:** 2020-02-24

**Authors:** Changmin Niu, Jiaqian Guo, Xueyi Shen, Shikun Ma, Mengmeng Xia, Jing Xia, Ying Zheng

**Affiliations:** ^1^ Department of Histology and Embryology School of Medicine Yangzhou University Yangzhou China; ^2^ Jiangsu Key Laboratory of Experimental & Translational Non‐coding RNA Research Yangzhou University Yangzhou China

**Keywords:** Cdl4‐Clu4A‐Ddb1 ubiquitinated degradation axis, cell cycle, PCNA, SETD8, spermatogenesis, stimulated by retinoic acid gene 8, transcriptional regulation

## Abstract

STRA8 (Stimulated By Retinoic Acid Gene 8) is a retinoic acid (RA) induced gene that plays vital roles in spermatogonial proliferation, differentiation and meiosis. The SETD8 and STRA8 protein interaction was discovered using the yeast two‐hybrid technique using a mouse spermatogonial stem cell (SSC) cDNA library. The interaction of these two proteins was confirmed using co‐immunoprecipitation and identification of key domains governing the protein: protein complex. STRA8 and SETD8 showed a mutual transcriptional regulation pattern that provided evidence that SETD8 negatively regulated transcriptional activity of the STRA8 promoter. The SETD8 protein directly bound to the proximal promoter of the STRA8 gene. STRA8 increased the transcriptional activity of SETD8 promoter in a dose‐dependent manner. For the first time, we have discovered that STRA8 and SETD8 display a cell cycle‐dependent expression pattern in germline cells. Expression levels of SETD8 and H4K20me1 in S phase of STRA8 overexpression GC1 cells were different from that previously observed in tumour cell lines. In wild‐type mice testis, SETD8, H4K20me1 and PCNA co‐localized with STRA8 in spermatogonia. Further, our studies quantitated abnormal expression levels of cell cycle and ubiquitination‐related factors in STRA8 dynamic models. STRA8 and SETD8 may regulate spermatogenesis via Cdl4‐Clu4A‐Ddb1 ubiquitinated degradation axis in a PCNA‐dependent manner.

## 1 INTRODUCTION

Spermatogenesis is a precise process regulated in both spatially and temporally dependent upon spermatogonial stem cells (SSCs). The proliferation and differentiation of spermatogonia require two rounds of meiosis of spermatocytes and spermatids which undergo spermiogenesis to become mature sperm. Studies on the functional mechanism of spermatogenesis‐related genes will lead to a greater understanding of spermatogenesis. These findings may provide a theoretical basis for gene regulation of spermatogenesis, offer molecular diagnosis methods and possible gene therapy for male infertility.

Retinoic acid (RA), an active metabolite of vitamin A in vivo, is a crucial signalling molecule for cell proliferation, differentiation, meiosis and sperm release.^1^, ^2^ Stimulated by Retinoic Acid Gene 8 (STRA8) is induced by RA and plays vital roles in spermatogenesis, primarily in spermatogonial proliferation, differentiation and meiosis. In the Han‐Chinese population, STRA8 mutation is associated with azoospermia and oligozoospermia.^3^, ^4^, ^5^, ^6^ Male STRA8 knockout (KO) mice are infertile, displaying a lack of meiotic and post‐meiotic germ cells. As a specific meiosis gatekeeper, STRA8 displayed a stage and cell specificity expression pattern in male spermatogenesis.^7^, ^8^ However, the molecular mechanism of STRA8 in spermatogenes remains unclear.

In a previous study,^9^ we found lysine methyltransferase 5A (SETD8), a sole enzyme known to specially catalyse monomethylation of Histone H4 Lysine 20 (H4K20me1), interacted with STRA8. STRA8 protein was used as bait to screen for interacting proteins from the mouseSSC cDNA library though the yeast two‐hybrid technique. SETD8 and H4K20me1 are integral to many key physiological processes, including transcriptional regulation,^10^, ^11^ cell cycle progression,^11^, ^12^, ^13^ DNA replication^14^ and regulation of PCNA^15^ to name a few. SETD8 was identified as a novel protein interacting with STRA8, the expression and function of which during spermatogenesis has not been previously studied.

In this study, we verified the protein‐protein interaction and determined the critical domains required for STRA8 and SETD8 interaction. The SETD8 protein directly binds to the proximal promoter of STRA8 gene and negatively regulates the transcriptional activity of STRA8. STRA8 increased the transcriptional activity of SETD8 promoter in a dose‐dependent manner. STRA8 is critical to the cell cycle transition from mitosis to meiosis; for the first time, our study revealed a STRA8 and SETD8 cell cycle‐dependent expression pattern in germ cells. In wild‐type testis, STRA8 co‐localized with SETD8, H4K20me1 and PCNA in spermatogonia. In STRA8 dynamic models, cell cycle‐related molecules and ubiquitination‐related factors displayed significant abnormal expression patterns. Our findings indicate that STRA8 and SETD8 may regulate spermatogenesis via Cdl4‐Clu4A‐Ddb1 ubiquitinated degradation axis in a PCNA‐dependent manner.

## 2 MATERIALS AND METHODS

### 2.1 Mice

Heterozygous B6.Cg‐STRA88^tm1Dcp/J^ mice (STRA8^+/−^ mice) were purchased from Jackson Laboratories (https://www.jax.org/) with a C57BL/6 genetic background. Homozygous (STRA8^−/−^, STRA8 KO) and wild‐type (WT) male mice were obtained from the mating pair of STRA8^+/−^ female and male mice. Two additional models were derived, a vitamin A deficient (VAD) male mouse model and a vitamin A recovery (VAR) male mouse model on C57BL/6 background.^16^ WT female and male mice were mated after being fed a vitamin A deficient diet for two weeks. The vitamin A deficient diet was maintained. The VAD mouse model was obtained after newborn mice were fed for 13‐14 weeks on the VAD diet. The VAD mice were then fed a normal vitamin A diet for 40 days to establish the VAD recovery mouse model. All animal feed was provided by the Trophic Animal Feed High‐tech Co., Ltd. All mice were maintained at the Laboratory Animal Center of Yangzhou University (Yangzhou, China). The day of birth was defined as 0 days post‐partum (dpp). All animal experiments of this study were approved by the Animal Ethics Committee of Yangzhou University.

### 2.2 Dual‐luciferase reporter (DLR) assay

We constructed mouse STRA8 promoter luciferase reporter plasmid by restriction digest with Xhol‐HindⅢ and SETD8 promoter reporter plasmids containing different fragments by restriction digest with KpnI‐HindⅢ. Respectively, they were named pGL3‐STRA8Pro, pGL3‐SETD8ProF1R (−1980~+1 bp), pGL3‐SETD8ProF2R (−1499~+1 bp), pGL3‐SETD8ProF3R (−1000~+1 bp) and pGL3‐SETD8ProF4R (−495~+1 bp). The primers sequences are provided in Table 1. Luciferase activity was quantified using the Dual‐Luciferase Reporter assay system (Promega). Firefly (Photinus pyralis) luciferase was used as an reporter gene and Renilla (Renilla reniformis) luciferase (pRL‐CMV) as the internal reference reporter gene. The specific fragment of the target promoter was inserted in front of the luciferase expression sequence. pGL3‐Basic were null reporter vectors without transcriptional activity and acted as a negative control. pGL4 luciferase vectors were used as positive controls as they contained numerous configurations. These included configurations with the synthetic Firefly Luc2 and Renilla hRluc luciferase genes, which have been codon optimized for more efficient expression and risk of anomalous transcription. Approximately 0.998 μg of reporter gene (pGL3‐Basic, pGL3‐target promoter or pGL4) was co‐transfected with 0.0014 μg pRL‐CMV into 293 cells and GC1 cells by Lipofectamine 2000 (Invitrogen) or 6 hours in a 24‐well plate. After 48 hours of continuous culture with complete media, the protein was collected. 100 μL of passive lysis buffer was added to each well to prepare the lysate of the transfected cells. Culture plates were frozen at −80°C for 10 minutes, and then rocked on a rocking platform at room temperature for 15 minutes. After centrifugation at 12000xg for 10 minutes, the supernatant was transferred to new tube for DLR assay. In DLR assay, the activities of Firefly and Renilla luciferases were measured sequentially from each single sample. The Firefly luciferase activity was measured first by adding 50 μL Luciferase Assay Reagent II to generate a stabilized luminescent signal and recorded as F. Renilla luciferase reaction was simultaneously initiated by adding Stop & GloR Reagent to the same tube and Renilla activity was recorded as F. According to the ratio of F/R, the activation degree of target reporter gene in different samples was compared. Luciferase activity is reported as the mean ± SEM of 3 experiments.

**Table 1:** Primer sequences used in this study

Name	Primer sequences	Name	Primer sequences
Stra8 Pro F	5′‐GGCTCGAGTGGAAACCCACAACGAAGG‐3′	Stra8(Myc)F1	5′‐GGGAGATCTCTATGGCCACCCCTGGAGAAG‐3′
Stra9 Pro R	5′‐GGAAGCTTGTCGCAGAATAAGAAGAGAGGC‐3′	Stra8(Myc)F2	5′‐GGGAGATCTCTAAAAAAGTCGATCTCTCCCAC‐3′
Setd8 Pro 1 F	5′‐GGGGTACCGTAGTCTTCGTTGTCCTGGAAC‐3′	Stra8(Myc)R1	5′‐GGGGCGGCCGCTTAAGCATCTGGTCCAACAGCCTC‐3′
Setd8 Pro 2 F	5′‐GGGGTACCCAAGCCTGCTTCAGGCTC‐3′	Stra8(Myc)R2	5′‐GGGGCGGCCGCTTACTCCTCCTCCTCTTCTTCTTCTTCC‐3′
Setd8 Pro 3 F	5′‐GGGGTACCGGAAGTCAGAGGACGATCTGC‐3′	Stra8(Myc)R3	5′‐GGGGCGGCCGCTTACAGATCGTCAAAGGTCTCC‐3′
Setd8 Pro 4 F	5′‐GGGGTACCGCAGTAAGCCTTTGCAACGTAG‐3′	RT‐Stra8 F	5′‐GCCGGACCTCATGGAATTTGA‐3′
Setd8 Pro R	5′‐GGAAGCTTGCTCAGCTAGACCGCAGC‐3′	RT‐Stra8 R	5′‐TCACTTCATGTGCAGAGATGATG‐3′
Stra8 Pro1 F	5′‐GTAAGAACTGGCGCTAGCC‐3′	RT‐Setd8 F	5′‐CAGACCAAACTGCACGACATC‐3′
Stra8 Pro1 R	5′‐CTGCGAACCAATCACAGC‐3′	RT‐Setd8 R	5′‐CTTGCTTCGGTCCCCATAGT‐3′
Stra8 Pro2 F	5′‐AGTCCACCTTTAAGGTTCTC‐3′	RT‐Pcna F	5′‐TTGCACGTATATGCCGAGACC‐3′
Stra8 Pro2 R	5′‐CTAGCGCCAGTTCTTACAC‐3′	RT‐Pcna R	5′‐GGTGAACAGGCTCATTCATCTCT‐3′
Stra8 Pro3 F	5′‐ACTGATGTGGTGGTGTCAG‐3′	RT‐cyclin A2(Ccna2) F	5′‐GCCTTCACCATTCATGTGGAT‐3′
Stra8 Pro3 R	5′‐AGAGAGAGAGAGAGAGGATGTG‐3′	RT‐cyclin A2(Ccna2) R	5′‐TTGCTCCGGGTAAAGAGACAG‐3′
Stra8 Pro4 F	5′‐CTGGTGCTACCAAAGTCGTG‐3′	RT‐cyclin E2(Ccne2) F	5′‐TCAGCCCTTGCATTATCATTGAA‐3′
Stra8 Pro4 R	5′‐GAGCGCCACCATATCAAACT‐3′	RT‐cyclin E2(Ccne2) R	5′‐CCAGCTTAAATCTGGCAGAGG‐3′
Stra8 Pro5 F	5′‐GTGTAGCTGCTCTTTGGAC‐3′	RT‐Cul4A F	5′‐TCTCACAAAGTCTCCCCAACG‐3′
Stra8 Pro5 R	5′‐TCTGATGCTTAAGGTACAG‐3′	RT‐Cul4A R	5′‐AGGACGTAGGTTCGATCCAGA‐3′
Stra8 Pro6 F	5′‐GGGCAGGAGCTATGTTAC‐3′	RT‐DDB1 F	5′‐ACCGGACACTTTACTTCAGCG‐3′
Stra8 Pro6 R	5′‐TCCTTCCATACTTTGACTTC‐3′	RT‐DDB1 R	5′‐CAATCTTCCCGTACATTCCCAC‐3′
Setd8(HA)F1	5′‐GAGATCTCTATGGCTAGAGGCAGGAAG‐3′	RT‐Cdt1 F	5′‐CTGCCTGGATTGGACTCCTG‐3′
Setd8(HA)F2	5′‐GAGATCTCTGAAGGCATGAAGATTGATC‐3′	RT‐Cdt1 R	5′‐CAGTTGTACTCTTTGTTCGCTTG‐3′
Setd8(HA)F3	5′‐GAGATCTCTGGGAACGTTATACGAAGCG‐3′	RT‐Dtl F	5′‐AACCAGGTGATAAACATTCCATAGTGGGTT‐3′
Setd8(HA)R1	5′‐GGCGGCCGCGTGCTTCAGCCAGGGGTAG‐3′	RT‐Dtl R	5′‐GACTGAAGAACGGGTCGTGGCAG‐3′
Setd8(HA)R2	5′‐GGCGGCCGCTTCCTTCCCGCTCTCAATC‐3′		
Setd8(HA)R3	5′‐GGCGGCCGCGGAGGCCTTGCTTCGGTCC‐3′		

### 2.3 Chromatin immunoprecipitation (ChIP) assay

A minimum of 4 × 10^6^ F9 cells were prepared for each chromatin immunoprecipitation. The following experiments were performed according to the instructions of SimpleChIP^®^ Enzymatic Chromatin IP Kit (Cell Signaling Technology). Formaldehyde and glycine were used to crosslink proteins to DNA, and 0.5 μL micrococcal nuclease per IP prep was used to digest DNA and 0.5 mol/L EDTA to stop digestion. Nuclei were completely lysed after 3 sets of 20‐seconds pulse sonication. After purification, DNA concentration was between 50 and 200 μg/mL and DNA was digested to a length of approximately 150‐900 bp. Each sample IP contained 5 to 10 μg of digested, crosslinked chromatin and an appropriate amount of antibody and incubated for 4 hours at 4°C with rotation. About 30 μL of Protein G Magnetic Beads was immediately added to each IP reaction and incubated for 2 hours at 4°C with rotation. After the elution of chromatin from antibody/protein G magnetic beads and reversal of crosslinks, DNA was purified using spin columns and analysed by quantitative reverse transcription PCR (qRT‐PCR) using specific primers (Table 1). IP efficiency was using the Percent Input Method using the Equation 1. With this method, signals obtained from each IP are expressed as a per cent of the total input chromatin. Absolute value: % Input: positive H3 > 1%, negative IgG < 0.1%, histone 1%‐50%, TF/cofactor: 0.2%‐1%.

Calculation of CHIP signal (%Input):

(1) PercentInput=2%×2(CT2%InputSample-CTIPSample)

CT = Threshold cycle of PCR reaction.

### 2.4 Cell culture and RA induction

The spermatogonia germ cell line (GC1 spg) was a gift from Professor Sha, State Key Laboratory of Reproductive Medicine, Nanjing Medical University, China. GC1 spg cell line was established by using SV40 large T antigen from 10 dpp BALB/c mouse testis. It is a cell type that intermediates between type B spermatogonia and pre‐leptotene spermatocyte. It exhibited some characteristics of early spermatogonia which did not express STRA8 but could be induced to express STRA8 by RA.^17^, ^18^, ^19^ The HEK‐239T cell line (Human embryonic kidney cells, lentivirus‐mediated overexpressed STRA8‐GC1 spg (SGC1) and its control GC1 spg (CGC1) were derived in our laboratory. Mouse P19 embryonic teratoma cell line (ZQ0200) and F9 testicular teratoma stem cell line (ZQ0199) were purchased from Zhong Qiao Xin Zhou Biotechnology Co.,Ltd. HEK‐ 293T, SGC1, CGC1 and F9 cells were maintained in complete media (Dulbecco's Modified Eagle Medium (DMEM) + 100 U/mL penicillin + 100 mg/mL streptomycin + 10% heat‐inactivated foetal bovine serum (FBS) (Zhong Qiao Xin Zhou)) and incubated at 37°C with 5% CO2 while P19 cells were maintained in MEMα(Zhong Qiao Xin Zhou) media. About 4 μmol/L RA (Sigma Chemical Co) was used to induce the expression of STRA8 for 24 hours in P19 and F9 cells.

### 2.5 Cell cycle synchronization

In order to further study the mechanism of interaction between STRA8 and SETD8, cell cycle synchronization studies for SGC1 and CGC1 were conducted. Cells were cultured under the treatment of serum starvation (without serum) for 72 hours to harvest G1 phase synchronized SGC1 and CGC1. Cells were cultured with media containing a final concentration 2.5 mmol/L thymidine for 14‐16 hours. The media was then changed to complete medium for 9 hours and then cultured within thymidine for 14‐16 hours again, finally in complete medium for 3 hours to achieve S phase synchronization. SGC1 and CGC1 were cultured in medium containing a final concentration 100 mg/L nocodazole for 8 hours to obtain M phase synchronization.

### 2.6 Construction of recombinant plasmids, cell transient transfection and immunoprecipitation (IP)

We constructed eukaryotic recombinant expression vectors containing mice STRA8 (1‐393aa) and different STRA8 fragments: STRA8F1R1(1aa‐142aa), STRA8F1R2 (1aa‐193aa) and STRA8F2R3 (194aa‐393aa). The target fragments and pCMV‐MYC vector were digested by restriction endonuclease of BgI II and Not I and then ligated. Target fragments SETD8 F1R1(1aa‐350aa), SETD8F1R2 (1aa‐213aa), SETD8F2R1 (214aa‐350aa), SETD8F2R3 (214aa‐341aa) and SETD8F3R2 (100aa‐213aa). The open reading frame (ORF) fragment of PCNA (140‐966bp) and pCMV‐HA vector were digested by EcoRI and XhoI and ligated. Primer sequences are provided in Table 1. After sequence analysis, these recombinants were transfected into cells for expression studies according to the Lipofectamine 2000 transfection reagent protocol (Life Technologies).

Following the method provided by the immunoprecipitation kit (Protein G) (Roche), the cells were lysed and then centrifuged to obtain the protein supernatant. 1~2 μg of antibody was added to 80 μL protein supernatant and incubated at 4°C for 4 hours on a rocking platform. Then, 80 μL of protein G was added and incubated at 4°C overnight on the rocking platform. The next day, the mix was washed and resuspended by the addition of 20‐30 μL 2× loading buffer. After samples were boiled, Western blot analysis was used to detect protein expression.

### 2.7 qRT‐PCR analysis

Total RNA was extracted by the isothiocyanate‐phenol‐chloroform method. Reverse transcription was performed according to the instructions of PrimeScript™ RT reagent Kit (Takara). qRT‐PCR analysis was performed following the method outlined by the GoTaq^®^ qPCR Master Mix kit (TaKaRa). Primer sequences are listed in Table 1. PCR thermal cycling conditions were carried out in two step RT‐PCR using a 10 μL reaction mix volume. The reactions were run in triplicate in a minimum of three independent experiments. Relative expression levels of the genes were calculated by cycle threshold (CT) values normalizing to GAPDH.

### 2.8 Western blot

Cells and tissue proteins were extracted using RIPA buffer. Protein extracts (50 μg) were subjected to SDS‐polyacrylamide gel electrophoresis and transferred to a polyvinylidene difluoride (PVDF) membrane which was activated by methanol. Post‐blocking with Tris‐Buffered Saline (TBS) containing 5% non‐fat milk for 1 hour at RT, the primary antibody was incubated using the anti‐sticking method (anti‐STRA8, 1:500; anti‐SETD8, 1:500, anti‐H4k20me1, 1:10 000) overnight at 4°C. The membranes were then incubated with secondary antibodies (ZSbio) at room temperature for an hour. Proteins were detected by ECL chemiluminescence.

### 2.9 Histology

Mice testes were fixed in Bouin's solution or 4% paraformaldehyde and dehydrated using a gradient alcohol series and embedded in paraffin for analysis. Sections (5‐μm thick) were prepared for haematoxylin and eosin (HE) staining and immunohistofluorescence following standard procedures. Cell types were identified by their location, morphological structure and chromatin.

### 2.10 Immunofluorescence

SGC1 and CGC1 cells were seeded onto 24‐well plates resulting in coverslips reaching 80%‐90% of cell density prior to immunofluorescence. Cells were fixed in 4% paraformaldehyde for 35 minutes, incubated with 50 mmol/L NH4CL for 10 minutes, washed with phosphate‐buffered solution (PBS) containing 0.2% triton and incubated with primary antibody at 4℃ overnight. Paraffin sections of mouse testes were dewaxed, and the antigen was repaired using a heating method. The sections were blocked with donkey serum at room temperature for an hour and then incubated using the primary antibody (Anti‐STRA8, custom made, 1:100; Anti‐SETD8, custom made, 1:100; anti‐H4K20me1, ab9051, Abcam, 1:250) at 4°C overnight. On the second day, secondary FITC‐labelled antibody (1:1000, Sigma) was incubated at 37ºC for an hour, and 5×6‐diamidino‐2‐phenylindole technique (DAPI, Solarbio) was used to label the nucleus. Images were obtained using a Nikon epifluorescence microscope.

### 2.11 Statistical analysis

Statistical analysis was performed using SPSS18.0 (SPSS Inc). Data are expressed as mean ± standard deviation (SD) and Student's *t* test. All experiments were repeated independently a minimum of three times. *P* value < .05 represents a statistically significant difference.

## 3 RESULTS

### 3.1 Mutual transcriptional regulation of STRA8 and SETD8

**Figure 1:**
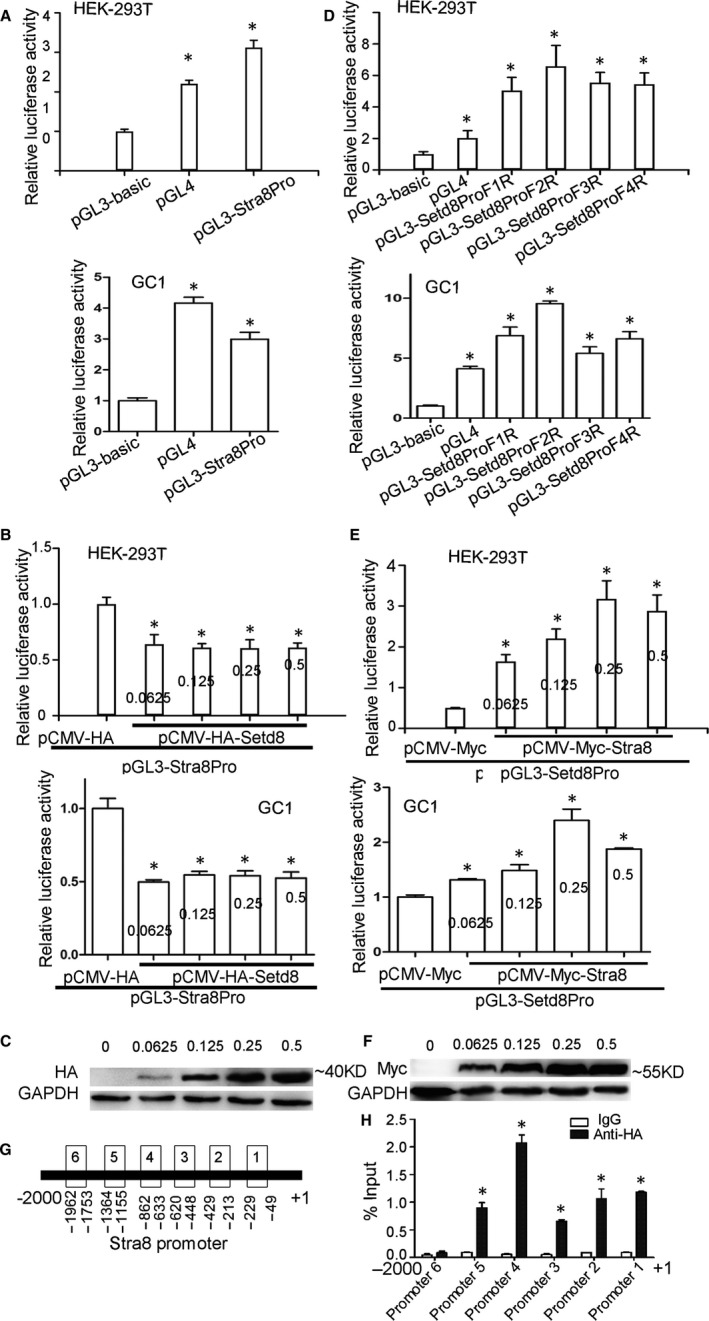
SETD8 repressed STRA8 expression by directly binding to the proximal STRA8 promoter. STRA8 increased the transcriptional activity of SETD8 promoter in a dose‐dependent manner. A, Transcriptional activity analysis of STRA8 promoter by DLR assay. pGL3 was a negative control group. pGL4 was a positive control group. B, Effects of SETD8 protein (pCMV‐HA‐SETD8, μg) with different doses on transcriptional activity of STRA8 promoter. C, Validation of SETD8 protein expression by Western blot. D, Transcriptional activity analysis of SETD8 promoter. E, Effects of STRA8 protein (pCMV‐MYC‐STRA8) with different doses on transcriptional activity of SETD8 promoter. F, Validation of STRA8 protein expression by Western blot. G, Schematic representation of primers structure of STRA8 promoter for ChIP assay. H, ChIP assay using anti‐HA antibody and control IgG. qRT‐PCR with specific primers was used to calculate the IP efficiency. The data were presented as mean ± standard deviation, * represented a significant statistical difference versus the control group, *P* < .05

Previously, we have reported the SETD8 and STRA8 protein interaction, but the mechanism of how this protein: protein combination may regulate inter‐transcriptional regulation during spermatogenesis remains unknown. To examine the transcriptional regulation of SETD8 on the STRA8 promoters, we co‐transfected the pCMV‐HA, pCMV‐HA‐SETD8 with the recombinant luciferase reporter plasmid pGL3‐STRA8Pro into HEK‐293T and GC1 spg, respectively, finding that the luciferase activity of the SETD8 eukaryotic expression plasmid was significantly lower than that of the pCMV‐HA plasmid transfected group (*P* < .05). We then varied the quantity of eukaryotic expression plasmid pCMV‐HA‐SETD8, 0.0625 μg, 0.125 μg, 0.25 μg and 0.5 μg, which were added into the pGL3‐STRA8Pro transfection group. We found different concentrations of pCMV‐HA‐SETD8 had no obvious affect on STRA8 promoter activity (Figure 1A,B). Western blot results verified that the expression of SETD8 protein increases with DNA concentration (Figure 1C). These results suggest that SETD8 protein inhibits the transcriptional activity of the STRA8 promoter but not in a dose‐dependent manner.

Subsequently, we constructed reporter plasmids containing different length fragments of the SETD8 promoter. Luciferase analysis demonstrated that all these SETD8 promoters had luciferase activity, and the promoter located upstream of SETD8 (−1499～+1 bp, F2R) reported the strongest transcriptional activity. From these studies, we concluded the SETD8 promoter F2R would be an ideal candidate for subsequent experiments (Figure 1D). pCMV‐MYC‐STRA8 and pGL3‐SETD8 ProF2R were co‐transfected into HEK‐293T and GC1 cells. Luciferase activity of STRA8 eukaryotic expression plasmid was significantly higher than that of pCMV‐MYC plasmid transfection group (*P* < .05). We then scaled the DNA concentration of pCMV‐MYC‐STRA8 0, 0.0625 μg, 0.125 μg, 0.25 μg and 0.5 μg, respectively. These studies found that the SETD8 promoter activity was significantly increased (*P* < .05) when the dose of pCMV‐MYC‐STRA8 increased, especially, at 0.25 μg and 0.5 μg plasmid concentrations (Figure 1E). Western blot analysis confirmed the expression of STRA8 protein was increased as DNA concentration ramped up (Figure 1F). These results suggest that STRA8 protein enhances the transcriptional activity of SETD8 promoter in a dose‐dependent pattern. Taken together, the above studies indicate that STRA8 and SETD8 are involved in spermatogenesis by mutual transcriptional regulation.

### 3.2 SETD8 directly binds to the promoter of STRA8

Deficient levels of SETD8 lead to embryonic lethality,^20^ while the absence of STRA8 results in no abnormalities except for reproductive defects.^16^ Knockout phenotypes indicate that SETD8 might be an upstream regulator of STRA8. To verify this hypothesis, F9 cells line that express endogenous STRA8 protein was used in ChIP assays. Using previous studies and analysis of promoter binding domain‐related sequences,^21^, ^22^ we designed six pairs of primers at −2000~−1 bp of the regulatory region of the mouse STRA8 gene as follows: promoter primer 1 (−49~−229 bp) contained DMRT1bs and RARE(DR2) (TGGGGTGAAAAGGTCA) motif, primer 2 (−213~−429 bp) contained DMRT1bs (TCCTTGAAA) motif, primer 3 (−448~−620 bp) contained Ebox3 motif (CATCTG), primer 4 (−633~−862 bp) contained Ebox1 motif (CAGCTG), Ebox2 motif (CAAGTGA) and RARE(DR4) motif (AGCTCACCTCAGGTCA), primer 6 (−1753~−1962 bp) contained TATA BOX (TATT) motif and primer 5 (−1155~−1364 bp) (Figure 1G). CHIP DNA samples and input DNA samples were analysed by qRT‐PCR. Calculation of CHIP signal (%Input) revealed that SETD8 could directly bind to the region −49~−229 bp, −213~‐429bp, −448~−620 bp, −633~−862 bp and −1155~−1364 bp of the STRA8 promoter (Figure 1H). These results show that the SETD8 protein directly binds to the proximal promoter of STRA8 to regulate its transcription.

### 3.3 Key interaction domains between STRA8 and SETD8

In order to investigate the mechanism of interaction between STRA8 and SETD8, we constructed different fragments of STRA8 and performed self‐activation activity analysis in our previous work. These studies identified a glutamic acid (GA)‐rich region (143‐193 aa) that had no self‐activation and toxicity. We reported that SETD8 acted as a interacting protein with STRA8, by yeast two‐hybrid technique in a mouse SSC cDNA library.^9^

**Figure 2:**
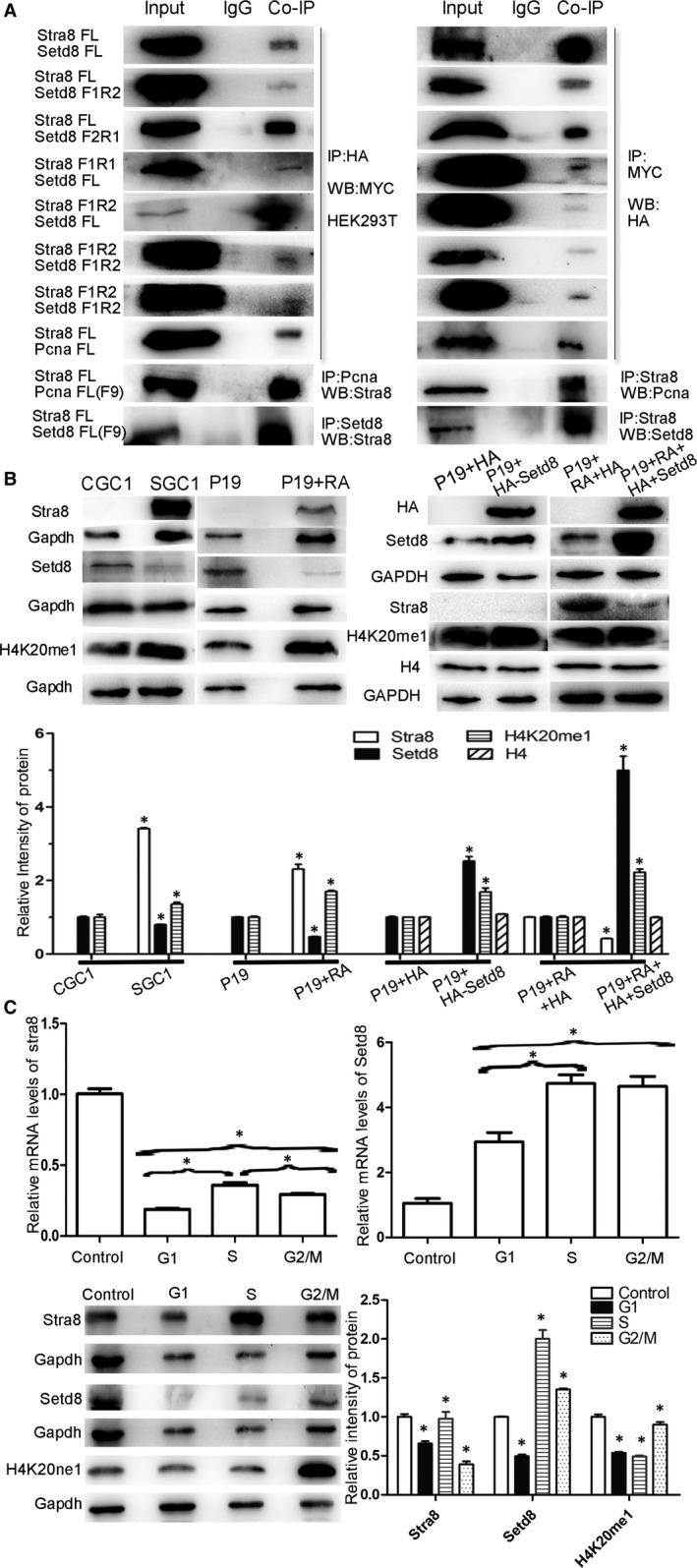
A, STRA8 interaction with SETD8 and PCNA in HEK‐293T and F9 cell lines. Domain of interactional between STRA8 and SETD8 was determined by CoIP. B, Expression analysis of STRA8, SETD8 and H4K20me1 in germ cells. P19 cells were induced to express endogenous STRA8 with 4 μmol/L RA. SETD8 protein was overexpressed by transient transfection. *P < .05, represented a significant statistical difference. C, RT‐PCR and Western blot analysis of STRA8 and SETD8 in cell cycle synchronized germline cells (*P < .05)

Consistent with the results of the yeast two‐hybrid experiments, the interaction between STRA8 and SETD8 was confirmed by co‐immunoprecipitation (CoIP) in transfected HEK‐293T cells and F9 cells which express endogenous STRA8 (Figure 2A). Recombinant eukaryotic expression vectors containing different SETD8 fragments were subsequently transfected into 293T cells, and the expressed proteins were identified by Western blot. Recombinant plasmids were co‐transfected with pCMV‐MYC‐STRA8 into 293T cells, respectively, and CoIP experiments conducted. SETD8 recombinants (F1R2 and F2R1) interacted with STRA8, while the other recombinants did not interact with STRA8 (Figure 2A).

Eukaryotic expression vectors containing different length mouse STRA8 protein fragments expression were confirmed using Western blot analysis all fragments expressed. Recombinant plasmids and pCMV‐HA‐SETD8 were co‐transfected into 293T cells. CoIP results confirmed that STRA8 recombinants (F1R1 and F1R2) could interact with SETD8 (Figure 2A). In addition, both truncated fragments F1R2 and F2R1 of SETD8 could interact with truncated fragment F1R2 of STRA8, but neither SETD8 F1R2 nor F2R1 could interact with STRA8 truncated fragment F1R1(Figure 2A).

### 3.4 Expression of STRA8 and SETD8 in cell cycle of germ cell line

SETD8 plays an essential role in cell cycle progression, the loss of which leads to cell cycle arrest in both humans and mice.^11^, ^12^ However, the cell cycle expression pattern of SETD8 in germ cell lines has not yet been reported. To establish if SETD8 has a cell cycle‐dependent expression pattern in germ cells similar to that of STRA8 cell cycle process, studies were performed in germ cells.

To confirm whether STRA8 and SETD8 are likely to interact during cell cycle progression or transcriptional regulation expression levels of SETD8 in germ cell lines, studies were conducted. The mRNA and protein levels of SETD8 were significantly decreased in the SGC1 group compared with the CGC1 group; conversely, the expression of H4k20me1 increased in the SGC1 group compared with that seen in the CGC1 group (Figure 2B). Results consistently showed that in P19 cell line, STRA8 was not expressed in P19 cells, but it could be induced by RA using a final concentration of 4 μmol/L. Expression levels of SETD8 were examined in P19 cells with the presence or absence of STRA8. These studies found that the expression of SETD8 was decreased and H4K20me1 increased in the RA induced P19 cells (Figure 2B). SETD8 protein was overexpressed by transient transfection, STRA8 protein expression was significantly decreased and H4K20me1 increased. However, overexpression of SETD8 alone in P19 cells could not induce the expression of STRA8 (Figure 2B). These results confirm the cooperative regulation between STRA8 and SETD8 in cell lines.

Cell cycle synchronization analysis was performed for the first time to confirm whether STRA8 and SETD8 operated in a cell cycle‐dependent expression pattern in germ cells. The cell cycle of SGC1 was synchronized at G0/G1, S, G2/ M phase, respectively, by serum deprivation, two times thymidine blocked and nocodazole. mRNA levels of STRA8 and SETD8 were the lowest in G0/G1 phase and gradually increased in S phase. But SETD8 mRNA maintained high levels of expression, while STRA8 mRNA decreased in G2/M phase. Compared with the untreated group, STRA8 mRNA levels of all phases were significantly decreased (*P* < .05) while SETD8 were all significantly increased (*P* < .05) (Figure 2B). The observation that total mRNA levels of STRA8 decreased and SETD8 increased under the same treatment indicated a negative regulation relationship between the proteins. Western blot analysis of G, S and M phase protein expression from synchronized SGC1 cells matched the observations of mRNA levels. Protein expression gradually increased in G1 and S phases and decreased in M phase, opposite to the protein expression levels of H4K20me1 which were lower in G1/S phase and peaked at M phase (Figure 2B).

**Figure 3:**
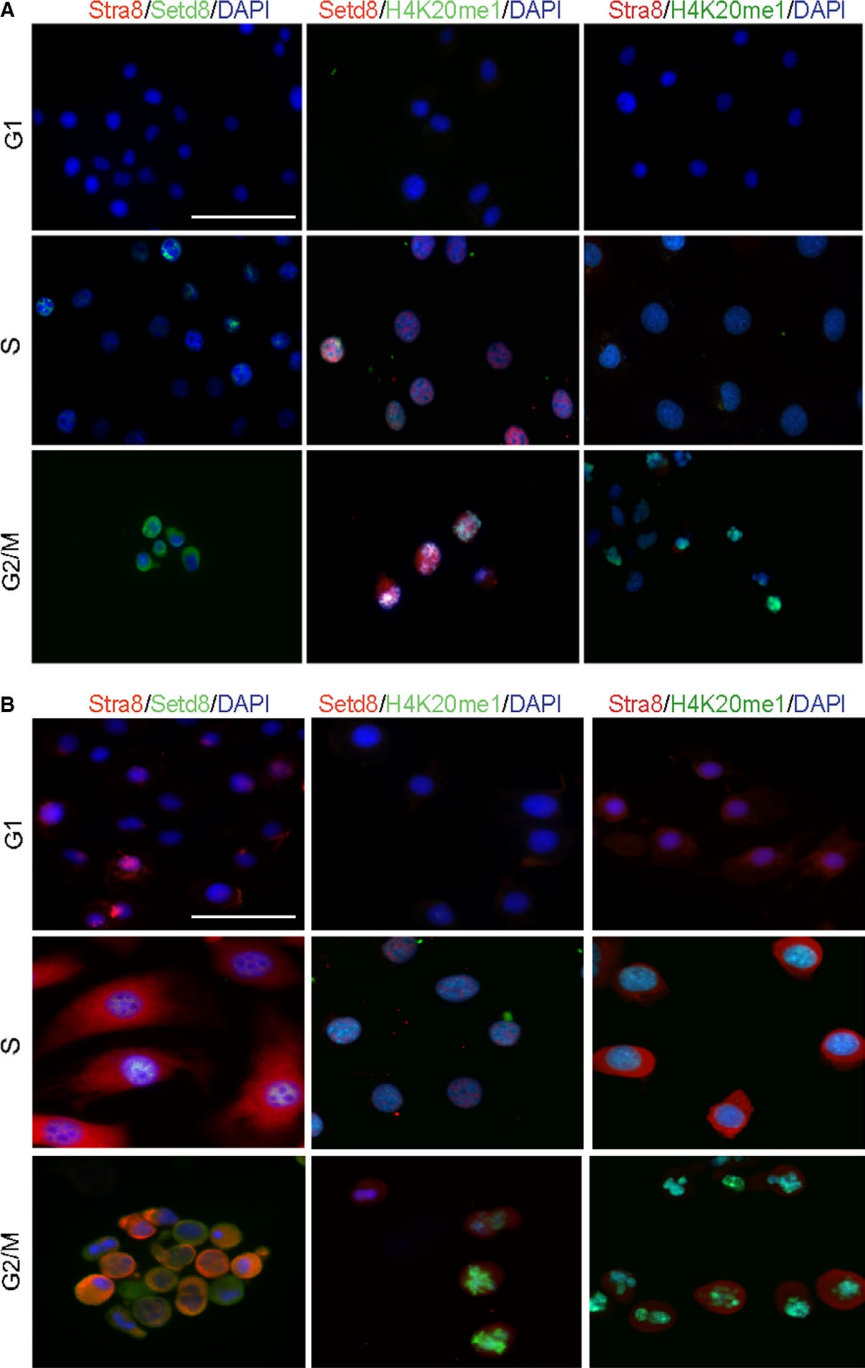
Immunostaining localization assay of STRA8, SETD8 and H4K20me1 in synchronized STRA8 overexpression germline cells. A, Localization of STRA8, SETD8 and H4K20me1 expression CGC1 cells, Scale bars = 200 μm. B, Localization of STRA8, SETD8 and H4K20me1 protein expression in SGC1 cells, Scale bar = 200 μm

Upon further examination, both mRNA and protein expression levels of STRA8 and SETD8 in different phases of cell cycle appeared to have a regular cycle. To further clarify the interaction mechanism of STRA8 and SETD8, immunostaining localization of STRA8, SETD8 and H4K20me1 in the synchronized germ cells was performed (Figure 3A,B). As previously reported, STRA8 is a cytoplasm and nuclear shuttle protein which is fully absent in CGC1 cells. In the synchronized SGC1 cells, the expression of STRA8 was weakly detected at G1 phase in cell nucleus, peaking at S phase both in the nucleus and cytoplasm. Subsequently, disappearing from the nucleus of M phase in the SGC1 cells while still weakly expressed in the cytoplasm, the expression of SETD8 and H4K20me1 was not detected in G1 phase synchronized CGC1 and SGC1 cells. SETD8 expression gradually increased in the nucleus during S phase but with lower quantity in SGC1 cells while the expression levels of H4K20me1 were elevated in SGC1cells compared with CGC1 cells in S phase. In M phase, SETD8 expression was high in both the cytoplasm and nucleus of CGC1 cells while quantities decreased in nuclear in SGC1 cells. H4K20me1 was only expressed in the nucleus but in high quantities of SGC1 cells. Subcellular localization analysis of STRA8, SETD8 and H4K20me1 in the synchronized cell cycle of SGC1 cells demonstrated that they also had a cell cycle‐dependent expression patterns in germ cells. Surprisingly, expression levels of SETD8 and H4K20me1 in S phase of SGC1 cells were different in tumour cells. These data are further demonstrated that STRA8 and SETD8 might regulate spermatogenesis through the germ cell cycle, but the mechanism still remains unclear.

### 3.5 Construction of STRA8 dynamic expression model

**Figure 4:**
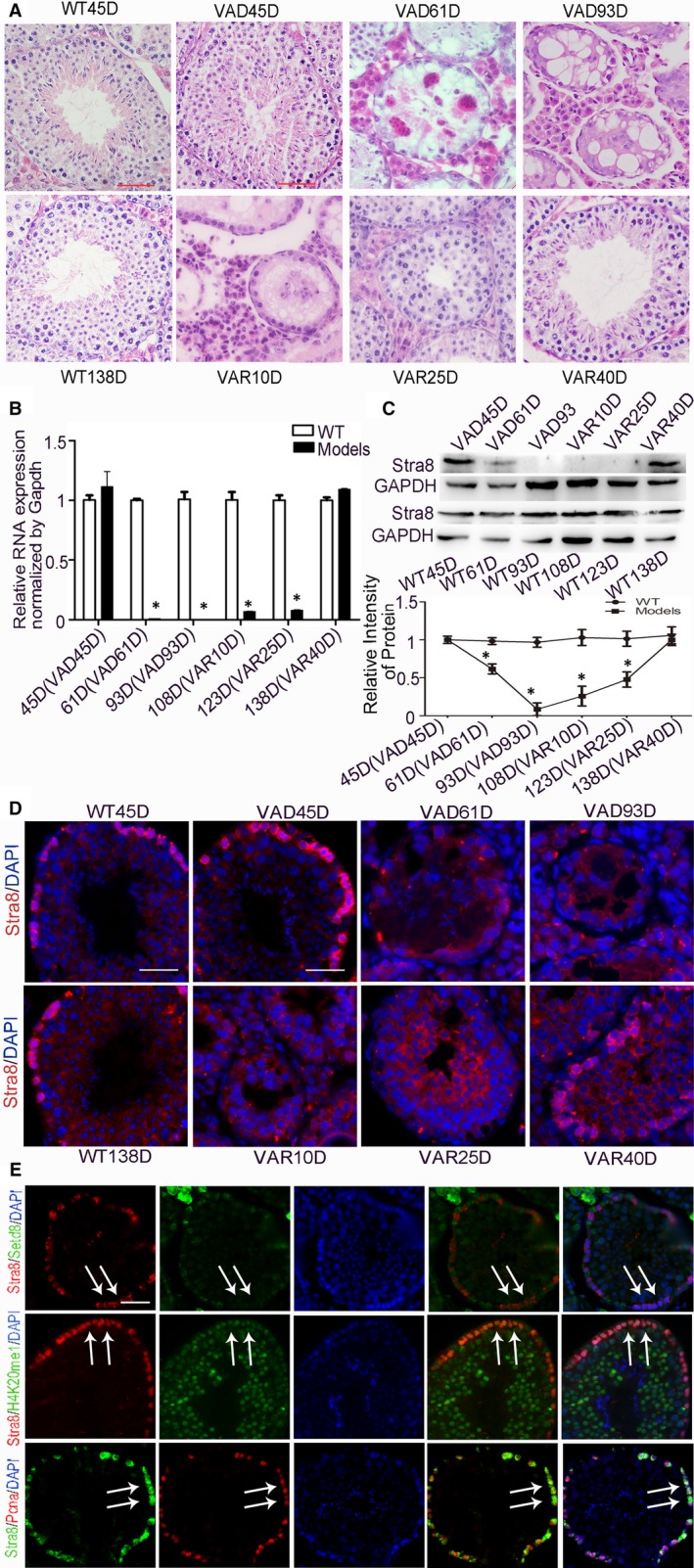
Construction of STRA8 dynamic expression mouse model. A, HE staining of VAD and VAR male mouse model. Scale bars = 50 μm. B, Relative mRNA expression analysis of STRA8 normalized to GAPDH, *represented statistical differences (*P < .05). C, Verification of the protein expression levels of STRA8 in the STRA8 dynamic expression mouse model. *P < .05 represented statistical differences. D, Immunostaining of STRA8 (red) and DAPI (blue) in WT testis and STRA8 dynamic expression mouse model. Scale bar = 50 μm. E, STRA8 co‐localized with SETD8, H4K20me1 and PCNA in adult mice testis. White arrows point to the co‐located positive germ cells. Scale bar = 50 μm

It has been shown in germ cells that SETD8 and STRA8 have similar cell cycle‐dependent expression patterns. A STRA8 dynamic expression model was constructed (VAD and VAR male mouse model) to study the mechanism of action of SETD8 and STRA8 in spermatogenesis (Figure 4). The testis from VAD 45D mouse showed a normal weight, and all phases of germ cells were arranged orderly within the seminiferous tubules. With prolonged vitamin A deficiency, vacuolization appeared at the basement of seminiferous tubules. Germ cells arrangement became disordered and reduced in quantity. After 61 days of VAD, all seminiferous tubules were filled with vacuoles and a significant reduction in diameter was observed; only a single layer germ cells remained. Spermatogenic process was severely impaired after 14 weeks of vitamin A deficiency. Only Sertoli cells and undifferentiated spermatogonia were present in the seminiferous tubules resulting in a complete loss of fertility. These results indicated the successful establishment of the VAD mouse model. Ten days post after resuming a normal vitamin A diet, the number of spermatogonia increased at basement of seminiferous tubules. After 25 days of a normal vitamin A diet, seminiferous tubules and spermatogonia arrangement returned to normal ordered distribution, and round sperm cells appeared. Germ cells at all phases were orderly arranged with normal fertility at 40 days after recovery. These data confirm the successful establishment of a VAR mouse model (Figure 4A).

We further analysed the STRA8 expression pattern in the model testis of VAD 45 days (45D), 61D and 93D and VAR 10D, 25D and 40D. There was no significant difference in the mRNA and protein expression levels of STRA8 in VAD45D testis compared with the WT group. STRA8 mRNA expression levels in VAD61D were undetectable, and protein quantities were low and ultimately undetectable. After vitamin A supplementation, the mRNA and protein expression levels of STRA8 gradually increased in model group and eventually reached WT levels in the testis by VAR40D (*P* > .05) (Figure 4B,C). The expression of STRA8 was analysed by immunostaining. In WT testis, STRA8 expression was elevated in the nucleus of spermatogonia and minimally expressed in the cytoplasm of early spermatocytes. STRA8 localization in VAD45D testis was similar to WT levels. At VAD61D, STRA8 was expressed only in the cytoplasm of spermatogonia and undetectable at VAD93D. STRA8 expression was detected in the cytoplasm of germ cells 25 days after vitamin A supplementation. STRA8 re‐expressed in spermatogonia and early spermatocytes at VAR40D which was consistent with WT mice (Figure 4D). Overall, we successfully constructed a STRA8 dynamic expression model, in which STRA8 expression levels were decreased by the deficiency of vitamin A and increased when vitamin A recovered. These analyses provide a foundation for further studies on the mechanism of interaction between STRA8 and SETD8 during spermatogenesis.

### 3.6 Expression of SETD8 and H4K20me1 in STRA8 dynamic models

In the testis of adult WT mice, STRA8 was expressed in spermatogonia and pre‐leptotene spermatocytes. SETD8, in WT testis, was highly expressed in Sertoli cells and Leydig cells, and expression was also observed in spermatogonia, spermatocytes and spermatozoa. H4K20me1 was highly expressed in spermatogonia, spermatocytes and spermatozoa of WT testis. PCNA was expressed in proliferating spermatogonia of WT testis. Immunofluorescence co‐localization studies showed that STED8, H4K20me1 and PCNA could co‐localize with STRA8 in spermatogonia (Figure 4E).

**Figure 5:**
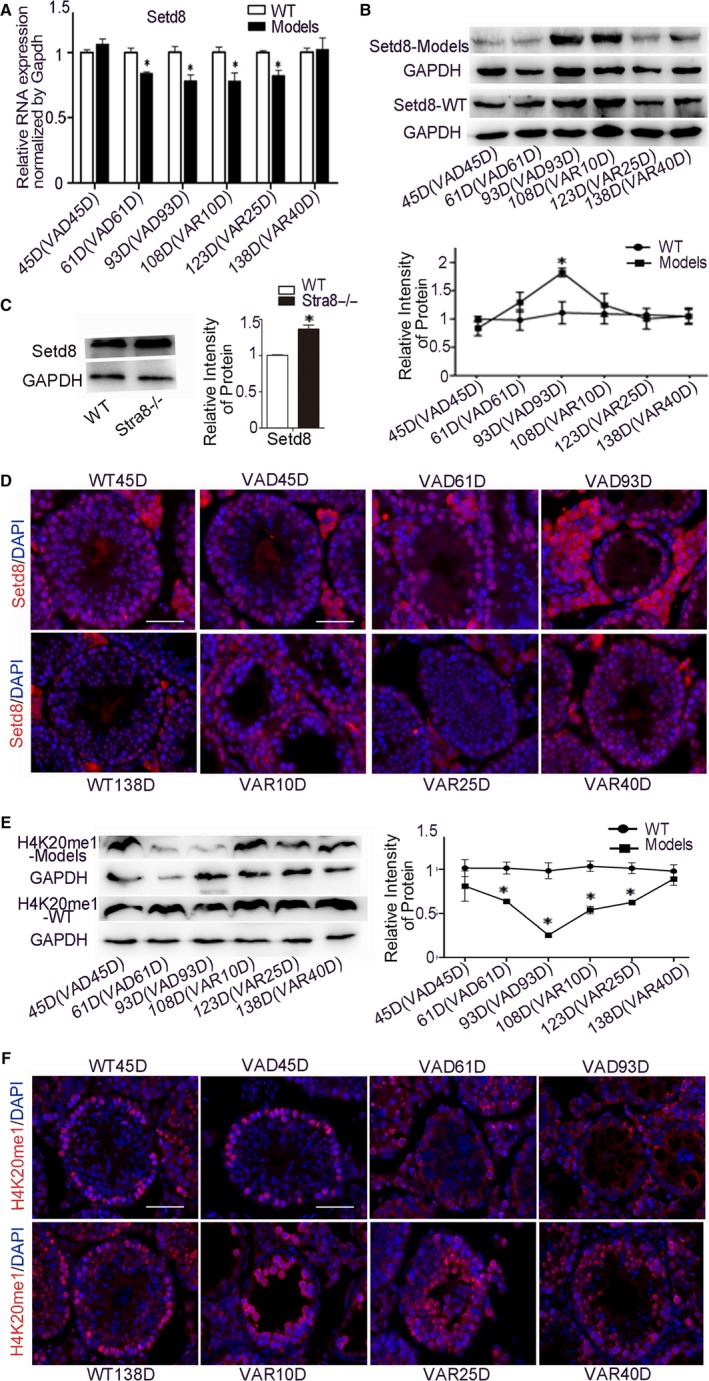
Expression analysis of SETD8 and H4K20me1 in VAD and VAR mouse models A, Relative mRNA expression analysis of SETD8 in WT, VAR and VAD mouse models. *P < .05 represented statistical differences. B, SETD8 protein expression analysis in VAD and VAR mouse models by Western blot. C, SETD8 protein expression analysis in STRA8 knockout testis. *P < .05 represented statistical difference. D, Immunofluorescence assay of SETD8 (red) and DAPI (blue) in WT testis and STRA8 dynamic expression mouse model. Scale bar = 50 μm. E, H4K20me1 protein expression analysis in STRA8 knockout testis. *P < .05 represented statistical difference. F, Immunofluorescence assay of H4K20me1 (red) and DAPI (blue) in WT testis and STRA8 dynamic expression mouse model. Scale bar = 50 μm

To further explore the mechanism of interaction between STRA8 and SETD8, we examined the expression patterns of SETD8, H4K20me1 and PCNA in the STRA8 dynamic expression models. The mRNA level of SETD8 decreased with the reduction in vitamin A and increased when returned to a normal vitamin A diet (Figure 5A). However, the protein expression of SETD8 showed an opposite expression pattern. SETD8 expression was significantly increased during the prolonged period of vitamin A deficiency and rapidly returned to normal levels after vitamin A recovery. These results were obtained from wild‐type testis. The expression of STED8 was significantly increased in 11 dpp STRA8 knockout testis which is consistent with that in STRA8 dynamic models (Figure 5B,C). The opposite expression pattern was observed for H4K20me1 protein (Figure 5E).

The subcellular localization of SETD8 was examined in different stages of the model group testes. During different stages of WT mice development, SETD8 was expressed in the nuclei of spermatogonia, spermatocytes, round spermatids and Sertoli cells (Figure 5D). H4K20me1 is involved in spermatogenesis with a similar distribution pattern with SETD8 (Figure 5F); the only observed difference was H4K20me1 high expression in spermatogonia and spermatids in stage IX‐XII. In the testis of VAD mice, even with the quantity of germ cells decreased significantly, SETD8 was highly overexpressed in the remaining spermatogonia and Sertoli cells. As spermatogenesis gradually recovered, SETD8 expression rebounded in spermatogonia, spermatocytes, round spermatid and Sertoli cells. However, H4K20me1 was expressed only in the cytoplasm of spermatogonia at VAD61D and 93D. There was no observed expression difference of H4K20me1 in WT and VAR testis.

### 3.7 Interaction studies between STRA8 and SETD8 during Spermatogenesis

**Figure 6:**
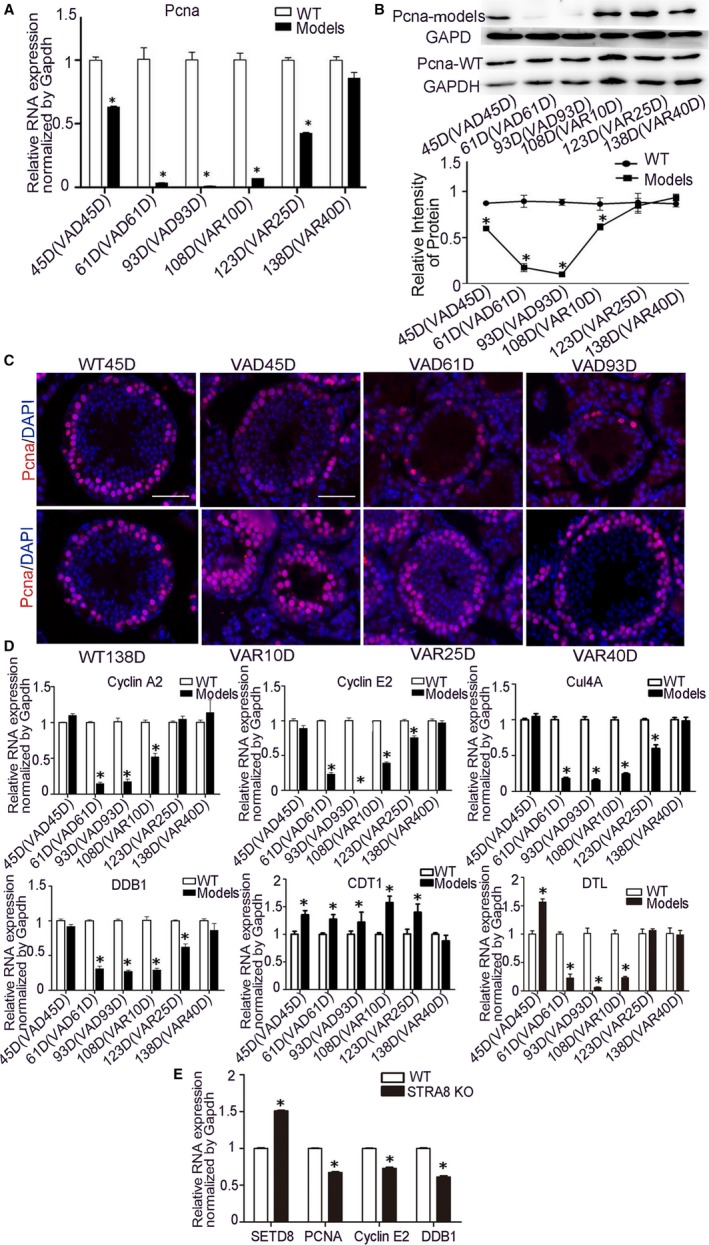
Expression analysis of PCNA, cell cycle‐related molecules and ubiquitination‐related factors in VAD and VAR models. A, Relative mRNA expression analysis of PCNA in WT and models. *P < .05 represented statistical differences. B, PCNA protein expression analysis in VAD and VAR models by Western blot. C, Immunofluorescence assay of PCNA (red) and DAPI (blue) in WT testis and STRA8 dynamic expression model. Scale bar = 50 μm. D, Relative mRNA expression analysis of Cyclin A2, Cyclin E2, Cul4A, Ddb1, Cdt1 and Dtl in WT and models by qRT‐PCR. *P < .05 represented statistical differences. E, Relative mRNA expression analysis of SETD8, PCNA, Cyclin E2, DDB1 in 11dpp WT and STRA8 KO testis by qRT‐PCR. *P < .05 represented statistical differences

We have demonstrated that STRA8 and SETD8 play a role in regulating the cell cycle of germ cell lines in vitro. It remains unclear whether the function of SETD8 during the cell cycle is affected by H4K20me1, PCNA or other cell cycle regulating molecules during spermatogenesis. To further investigate how STRA8 and SETD8 interact with each other during spermatogenesis, the expression of the SETD8‐interacting protein PCNA in STRA8 dynamic expression models was measured (Figure 6A‐C).

Both mRNA and protein levels of PCNA fluctuated with changes in STRA8 levels but recovered faster than that of STRA8 when vitamin A was supplemented. Using immunohistofluorescence studies, the majority of PCNA expression was localized to the nucleus of spermatogonia and spermatocytes in the germ cells from WT testis. However, with prolonged vitamin A deficiency, the expression of PCNA was significantly affected. Approximately only three PCNA positive cells were counted in each of the seminiferous tubule of VAD93D testis. However, after initiation of a normal vitamin A diet, the expression of PCNA quickly returned to normal levels. There was no significant difference in the expression of PCNA between VAR40D and WT mice testes. The subcellular localization of PCNA studies further confirmed mRNA and protein expression patterns.

Previous studies have reported that SETD8 protein levels require precise regulation using many regulatory routes to appropriately regulate cell cycle progression and its degradation in S phase is dependent on ubiquitination and phosphorylation. For these reasons, the expression levels of cell cycle‐related molecules Cyclin A2 (Ccna), Cyclin E2 (Ccne2) and ubiquitination‐related factors Ddb1 (damage specific DNA binding protein 1), Cul4A, Cdt1 (chromatin licensing and DNA replication factor 1) and Dtl (denticleless E3 ubiquitin protein ligase) were analysed in STRA8 dynamic mouse models. The mRNA levels of Cyclin A2, Cyclin E2, Ddb1, Cul4A and Dtl were changed consistently with STRA8, while Cdt1 displayed an opposite trend in these mouse model studies (Figure 6D). In addition, we performed RNA‐sequencing in a previous study on STRA8 KO and WT testis at 11 dpp, the time that no significant differences existed in morphology and number of germ cells of WT and KO testis. The differentially expressed genes detected by RNA‐seq included SETD8, PCNA, Cyclin E2 and DDB1, which were the same as those detected in STRA8 dynamic model. qRT‐PCR was performed to verify the sequencing results. Compared with WT mice, the expression level of SETD8 was significantly increased in KO testis while PCNA, Cyclin E2 and DDB1 were significantly decreased. The expression trends of above molecules are consistent with what we detected in STRA8 dynamic model (Figure 6E).

## 4 DISCUSSION

Spermatogenesis is a complex process involving precise regulation of cell division and differentiation. Previous studies have shown the STRA8:SETD8 interaction, both of which may act as transcription factors to regulate the functions of other genes.^10^, ^11^, ^23^, ^24^ SETD8 knockout mice are embryonic lethal,^20^ whereas STRA8 knockouts show no abnormal phenotype beyond reproductive deficiency.^16^ These different phenotypes indicated SETD8 might be an upstream regulator of STRA8. The studies presented here sought to examine a transcriptional regulation analysis to clarify the STRA8:SETD8 transcriptional relationship. Discovering that SETD8 does negatively regulate transcriptional activity of the STRA8 promoter to directly affect the function of STRA8, additional studies are needed to explore whether H4K20me1 is involved in the transcriptional regulation of STRA8. Interestingly, STRA8 increased the transcriptional activity of the SETD8 promoter in a dose‐dependent manner. ChIP assay experiments concluded that the SETD8 protein directly binds to the proximal promoter (−1364 bp) of STRA8 gene, particularly in the region containing Ebox1, Ebox2, Ebox3, DMRT1bs, RARE(DR2) and RARE(DR4) motif. Determining the domains required for the SETD8 protein to bind STRA8 requires further mutagenesis experiments. STRA8 and SETD8 shared a mutual transcriptional regulation pattern in both HEK‐293T cells and GC1 cells. Our evidence that STRA8 is downstream SETD8 in a signalling pathway, and STRA8 is a transcription factor that affects the SETD8 promoter activity. However, it is unclear how mutual transcriptional regulation between STRA8 and SETD8 contributes to the cell cycle transition.

In our previous work,^9^ SETD8 was identified as a STRA8 interacting protein. The interaction was verified by CoIP in transfected HEK‐293T cells and F9 cell lines which express endogenous STRA8. Four eukaryotic recombinant expression vectors were constructed containing different SETD8 fragments and three different STRA8 fragments to determine key protein:protein interaction domains. The STRA8 gene contains a GA‐rich region (143‐193 aa) that contains no self‐activation and toxicity while SETD8 contained a SET‐domain (226‐342aa). In this study, STRA8 F1R2 (1‐193 aa) but not F1R1(1‐142 aa) did interact with truncated fragment F1R2 (1‐213 aa) and F2R1(214‐350 aa) of the SETD8 protein. Mutation of the GA‐rich region and SET‐domain is required to identify additional key sites required for the STRA8:STD8 protein interaction.

Mammalian spermatogenesis involves spermatogonia mitosis, spermatocytes meiotic division, pre‐meiotic DNA replication and spermiogenesis. Pre‐leptotene spermatocytes underwent pre‐meiotic DNA replication before entering meiosis. While DNA replication completed, STRA8‐deficient pre‐leptotene spermatocytes failed to enter meiosis prophase. Within 24 hours following the pre‐meiotic S phase, numerous mutant spermatocytes displayed premature chromosome condensation and metaphase‐like topography with 40 univalent chromosomes, which similar to normal mitotic metaphases, followed by rapid apoptosis.^7^, ^8^, ^25^ Although these cells already had the morphological appearance of pre‐leptotene spermatocytes, they retained the proliferative characteristics of spermatogonia and tried to divide rather than enter the prophase of meiosis. The abnormal transition from the cell cycle process of mitosis to the meiosis might directly lead to spermatogenesis failure. STRA8 plays an important role in the transition from mitosis to meiosis in the cell cycle. For this reason, we used germ cell lines and STRA8 dynamic mouse expression models to further study the mechanism of interaction between STRA8 and SETD8 during spermatogenesis.

Bouillet P and Oulad‐Abdelghani M research group^26^, ^27^ isolated a number of novel RA‐responsive genes by using the P19 pluripotent embryonal carcinoma (EC) cell line and collectively designated as STRA genes (STRA1‐12). Expression pattern analysis of the STRA genes showed that only STRA8 exhibited a precise tissue‐specific and developmental manner, while other STRA genes were expressed in multiple tissues, but not in testis or at low levels. The STRA8 gene was specifically expressed in male mice testis and restricted to type B spermatogonia and pre‐meiotic germ cells. Although STRA8‐deficient testis initiated meiosis, we discovered aberrant expressions of Cyclin A2^28^, ^29^, ^30^, ^31^ and Cyclin E2.^31^, ^32^, ^33^, ^34^ Both of these cycle factors, which began to express in late G1 phase, peaked in S phase and began to decline in G2 phase. The expression of Cyclin A2 and Cyclin E2 played a role in the initiation of DNA synthesis to prevent DNA replication. Cyclin E2 combined with CDK2 to promote G1/S phase transition and primarily expressed in post‐meiotic spermatocytes. In STRA8‐deficient testis, the expression level of Cyclin E2 decreased significantly due to the loss of post‐meiotic germ cells. Cyclin A2 played a major role in S phase and was highly expressed in spermatogonia and pre‐leptotene spermatocytes. Theoretically, Cyclin A2 should be increased in STRA8‐deficient testis; however, we found Cyclin A2 was reduced in STRA8‐deficient testis. This evidence reveals the aberrant cell cycle in STRA8‐deficient testis, and possibly other important molecules are also involved in the regulation of the cell cycle during spermatogenesis. These dynamic changes in gene expression were caused by STRA8, but we could not rule out the roles of other STRA genes which exhibited low expression in the testis. We will explore whether STRA genes with low expression in the testis have influence on this process in later studies.

In STRA8 dynamic expression mouse models, SETD8, as an important regulator of cell cycle process and genome stability, was increased with the decrease of STRA8 expression. H4K20me1 fluctuated with STRA8 levels. These changes in expression levels were consistent with results seen in germ cell lines. In Hela cells, the expression level of SETD8 was the lowest in S phase, increased gradually in late S phase, and was higher in G2/M and G1 phase. The expression pattern of H4K20me1 was similar to SETD8.^11^, ^12^ It is not clear whether SETD8 has a germline cell cycle‐dependent expression and if STRA8 displays a similar expression pattern. For the first time, we studied the expression patterns of SETD8 and STRA8 in the germline cell cycle. Comparing between cell lines, the expression levels of SETD8 increased significantly in S phase, but no significant difference was observed in G2/M phase in germ cell lines, which were different from previously reported in literature. Most of the current research on SETD8 is focused on using cancer cells, while the work we presented here use germline cell lines. SETD8 might play different roles in germline cells versus cancer cells, leading to expression changes during different stages of the cell cycle. STRA8 had the lowest expression in G1 phase and reached peak in S phase but decreased significantly in G2/M phase. In STRA8 overexpressed GC1 cells, SETD8 expression decreased in S/M phase, while the quantity of H4K20me1 protein levels increased. Both in vitro and in vivo experiments showed that SETD8 and H4K20me1 expression levels were linked with STRA8, which provides evidence that STRA8 and SETD8 might regulate spermatogenesis during the cell cycle.

In the mammalian cell cycle, DNA replication is precisely regulated to ensure genomic integrity and stability. The formation of pre‐replication complexes (pre‐RCs) at the replication origins (ORIs) marks the beginning of DNA replication. The process of replication licensing occurred only once in each cell cycle all before S phase.

**Figure 7:**
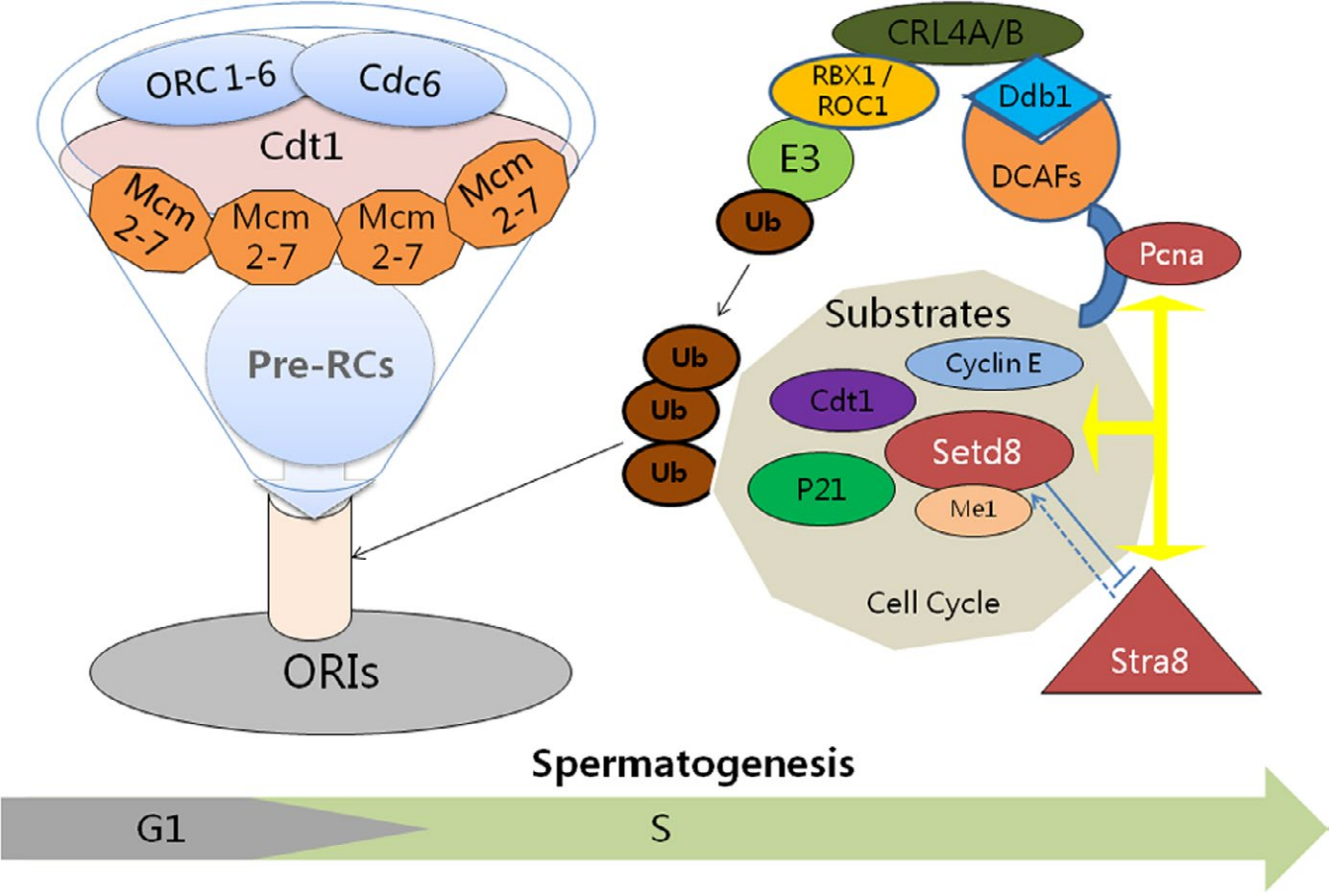
Meiotic gatekeeper STRA8 and SETD8 regulate spermatogenesis via Cdl4‐Clu4A‐Ddb1 ubiquitinated degradation axis in S phase by a PCNA‐dependent manner. Yellow arrow: SETD8 protein interacts with PCNA, regulating S phase progress of cell cycle. STRA8 protein can interact with SETD8 and PCNA protein, respectively. Blue solid line represents that SETD8 protein inhibits the promoter activity of STRA8 gene. Blue dotted line arrow represents STRA8 protein promotes the promoter activity of SETD8 gene

Pre‐RCs included ORC 1‐6, CDC6, CDT1 and MCM2‐7,^35^, ^36^, ^37^ ORC bound to CDC6 and then recruited MCM2‐7 to form the pre‐RC by interacting with CDT1, which assemble at ORIs to initiate DNA replication. CDT1 is a key regulator of replication licensing. CDT1 is highly expressed in G1 phase and degraded in S phase. Meanwhile, SETD8 and the monomethylation modifications of H4K20 were also directly involved in the assembly of pre‐RC on ORIs. CDT1 and SETD8 were degraded completely or maintained at minimal levels in S phase to prevent DNA replication.^38^, ^39^, ^40^ In our study, it was found that the expression of SETD8 decreased after the overexpression of STRA8 in spermatogonia. The expression of SETD8 and CDT1 increased significantly in juxtaposition compared with PCNA and H4K20me1 which decreased in the testis with STRA8 reduction. Expression patterns of CDT1, SETD8 and PCNA suggested that STRA8 might affect the formation of pre‐RC and its assembly on ORIs, leading to an abnormal state of G1/S phase and abnormal degradation of S phase. Current reports suggest that SETD8 needed to be maintained at a precise level to ensure appropriate cell cycle progression, including ubiquitin E3 ligase SCF/skp2,^14^ Cul4‐Ddb1,^41^ CRL4‐DTL,^31^, ^38^, ^40^, ^42^ APC‐cdh1 ubiquitin complex which mediated ubiquitination and CDK1/Cyclin B complex phosphorylation.^12^ In G1/S and S phase, the main degradation pathway of SETD8 was ubiquitination mediated by Cullin‐RING E3 ubiquitin ligase (CRL4‐E3 ubiquitin ligase).^43^ CRL4‐E3 ubiquitin ligase, a member of CRL family, consists of a ring finger domain protein RING‐box protein 1 (RBX1), scaffold protein cullin4 (CUL4‐CUL4A and CUL4) and DDB1‐CUL4‐related substrate receptor (DCAF). The expression levels of DTL (Cdt2, also known as DCAF2), CUL4A, DDB1 and PCNA in STRA8‐deficient testis were significantly decreased and returned to normal levels soon after STRA8 expression was restored. Cullin, as a scaffold protein, bound to RBX1/ROC1, DDB1 as a cohesion factor, bound to CUL4 and DCAFs. Abnormal expression of CUL4A and DDB1 in STRA8 deficient testis might directly affect the correct assembly of E3 ligase complexes. The CUL4A‐DDB1‐E3 ligase complex in conjunction with the substrate receptor DCAF2 degraded Cdt1 and SETD8 by ubiquitination in a PCNA‐dependent manner, preventing the re‐replication of DNA after S phase or DNA damage.^37^, ^44^ In WT testis, PCNA, as a known SETD8 interacting protein, co‐localized with STRA8, SETD8 and H4K20me1, suggesting that STRA8 and SETD8 might depend on H4K20me1 and PCNA to regulate the cell cycle during spermatogenesis. P21 inhibited the activity of Cdk2 kinase, thus preventing CDT1, SETD8 and P21 from entering or passing through S phase. The abnormal expression of CDT1, SETD8 and P21 after STRA8 reduction providing additional evidence that abnormal regulation of Cdt2‐Ddb1‐CUL4A E3‐ubiquitination degradation axis in S phase (Figure 7). The expression of SETD8 in germline cell cycle is influenced by many factors. The level of H4K20me1 varied with the changes in SETD8 and STRA8. However, it is not clear how H4K20me1 participates in the regulation of the STRA8:STD8 interaction. STRA8 promotes spermatogonial differentiation and is required for meiotic initiation. STRA8 co‐localized with STED8 in spermatogonia; however, it is unclear whether STRA8 and STED8 are required for differentiation or meiosis. They may interact in the meiosis initiation or spermatogonia differentiation, or both processes. In future studies, we will establish and culture spermatogonial stem cell lines (SSCs) to induce differentiation, which may help to determine the exact period of their interaction.

STRA8 is a RA induced gene. In juvenile C57BL/6 males lacking STRA8 gene function, the early mitotic development of germ cells appears to be undisturbed. However, these cells then fail to undergo the morphological changes that define meiotic prophase, and they do not display the molecular hallmarks of meiotic chromosome cohesion, synapsis and recombination. The Anderson EL group concludes that STRA8 regulates meiotic initiation in spermatogenesis.^7^ The Endo T group reported that undifferentiated spermatogonia accumulated in unusually high numbers as early as 10 d after birth in mice lacking STRA8, whereas differentiating spermatogonia was depleted. They thus conclude that STRA8 promotes (but is not strictly required for) spermatogonial differentiation.^3^ In summary, STRA8 promotes spermatogonial differentiation and is required for meiotic initiation.

STRA8 has been proved to be involved in the process that leads to stable commitment to the meiotic cell cycle but does not affect DNA replication in pre‐meiosis.^25^ In our study, STRA8, as a meiotic gatekeeper, co‐localized with STED8 in spermatogonia, is considered to regulate meiosis cell cycle by interacting with SETD8. We also found the differentially expressed genes related to spermatogonia differentiation in the sequencing results of STRA8 knockout mice,^45^ which further suggesting its role in differentiation. However, whether it is related to the role of SETD8 needs further verification. In the next step, we will establish and culture spermatogonial stem cell lines (SSCs) to induce differentiation, which may help to determine whether the interaction is required for differentiation. Fully understanding the molecular mechanism of STRA8 and SETD8 in spermatogenesis requires further study.

## 5 CONCLUSION

The interaction between SETD8 and STRA8 has been previously described. STRA8 and SETD8 showed a similar transcriptional regulation pattern. The SETD8 protein directly binds to the proximal promoter of the STRA8 gene. STRA8 and SETD8 expression patterns are cell cycle‐dependent in germline cells. STRA8 plays an important role in the transition from mitosis to meiosis cell cycle and co‐localized with SETD8, H4K20me1 and PCNA in WT spermatogonia. In STRA8 dynamic expression models, cell cycle‐related molecules and ubiquitination‐related factors displayed abnormal expression. STRA8 and SETD8 may regulate spermatogenesis via Cdl4‐Clu4A‐Ddb1 ubiquitinated degradation axis in S phase by a PCNA‐dependent manner. In this study, we explored the interaction between meiosis gatekeepers STRA8 and SETD8 during spermatogenesis and laid a foundation for further studies on the mechanism of interaction between them. The study of these spermatogenesis‐related proteins will lead to a further understanding of spermatogenesis, provide a basis for the gene regulation of spermatogenesis, and molecular diagnostic methods and gene therapy possibilities for male infertility.

## CONFLICT OF INTEREST

All authors declare no conflict of interest.

## AUTHOR CONTRIBUTIONS

CN and JG designed and carried out experiments. XS carried out cell experiments. SM built the mice models. MX and JX collected and analysed the data. YZ designed the study. All authors checked and approved the final manuscript.

## Data Availability

All data generated during the study are available from the corresponding author (Dr YZ) on request.
